# Direct observation of vibrational energy dispersal *via* methyl torsions

**DOI:** 10.1039/c7sc05309f

**Published:** 2018-01-24

**Authors:** Adrian M. Gardner, William D. Tuttle, Laura E. Whalley, Timothy G. Wright

**Affiliations:** a School of Chemistry , University of Nottingham , University Park , Nottingham NG7 2RD , UK . Email: Tim.Wright@nottingham.ac.uk

## Abstract

Explicit evidence for the role of methyl rotor levels in promoting energy dispersal is reported. A set of coupled zero-order vibration/vibration-torsion (vibtor) levels in the S_1_ state of *para*-fluorotoluene (*p*FT) are investigated. Two-dimensional laser-induced fluorescence (2D-LIF) and two-dimensional zero-kinetic-energy (2D-ZEKE) spectra are reported, and the assignment of the main features in both sets of spectra reveals that the methyl torsion is instrumental in providing a route for coupling between vibrational levels of different symmetry classes. We find that there is very localized, and selective, dissipation of energy *via* doorway states, and that, in addition to an increase in the density of states, a critical role of the methyl group is a relaxation of symmetry constraints compared to direct vibrational coupling.

## Abstract 2

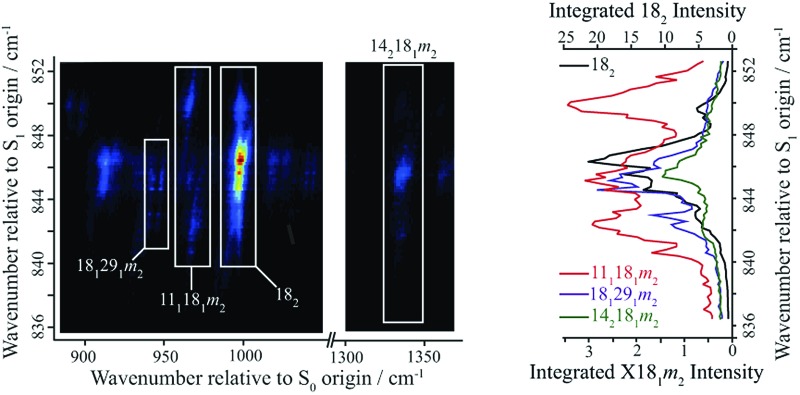
Explicit evidence for the role of methyl rotor levels in promoting energy dispersal is reported.

## 1. Introduction

The dispersal of energy through a molecule can be a valuable aid to increasing its stability following the input of localized energy, for example following photoexcitation or as the result of the formation of a chemical bond.^1^–^3^ The process of energy dispersal is often termed intramolecular vibrational redistribution (IVR) as studies are usually focused on vibrational coupling,^4^–^12^ but, following on from earlier work,^13^ recent studies have generalized this to include vibration-torsional coupling in cases where an internal rotor is present in the molecule.^14^–^17^ If energy cannot rapidly be dispersed, then the molecule may dissociate following photoexcitation, or a nascent chemical bond may simply break again in a biomolecular reaction. In the former case, the process is termed photodissociation and the resultant formation of radical products can affect the observed chemistry in a reactive system. In a biological context, this can be very harmful to the host organism as radical formation can precipitate cell damage and the initiation of cancer development.^18^ Of course, the ability of many naturally-occurring biomolecules to be able to deal with high levels of incident radiation is known, and organisms may possess other protection mechanisms, such as tanning in humans. The ability for us to be able to protect ourselves against high levels of solar radiation is becoming ever more important, and so the design of molecules that have photophysical characteristics that enable them to disperse energy quickly is important in the development of more-efficient sun protection products, for example.^18^–^20^ Although much ongoing work has focused on the coupling between electronic states *via* conical intersections, in either electronic state the delocalization of energy through the chemical bond network is also a key aspect^21^ and, as noted, the relevant motions are vibrations and, where they exist, torsions. In all cases, the critical aspect is the coupling between these modes, with anharmonicity and vibration-torsional coupling being the principal facilitators, although vibronic Herzberg–Teller (HT) interactions can be important. In addition, rotations can play a role *via* Coriolis effects,^9^,^10^ decoherence^6^,^22^ and torsion–rotation interactions.^23^

Uncovering general mechanisms and aspects of molecular structure that are important in promoting energy dispersal, is clearly important in being able to establish principles for the design of photolytically robust molecules.^20^ To this end, much work has been done on families of molecules, where a key aspect of the molecular structure is changed, and the effect on vibrational energy dispersal is ascertained. Such studies include the work on acetylenes by Field, Scoles and coworkers,^24^,^25^ monoalkylbenzenes by Saykally and coworkers,^26^ comparisons between *para*-difluorobenzene (*p*DFB) and *para*-fluorotoluene (*p*FT) by Parmenter and coworkers,^27^–^29^ and between toluene, toluene-α-*d*_3_ and *p*FT by Reid, Wright and coworkers.^16^,^30^ The work on substituted benzenes is particularly pertinent since many biological molecules contain a phenyl ring, which often acts as the chromophore in such species. As such, gaining insight into the photophysical behaviour of substituted benzenes, and observing the effect of varying the substituents, has the potential to give deep insight into the fundamental processes occurring in more-complicated biomolecules. Of particular interest is the observation that methyl substitution appears to affect the photophysics of molecules, both excited electronic state lifetimes and the flow of vibrational energy through a molecule. Understanding the details of such results, and also unpicking intramolecular *versus* solvent-induced phenomena, is key to understanding, for example, the different behaviour of uracil and thymine,^31^,^32^ with the latter being a methylated version of the former. Despite much attention, the role of the methyl group is still unclear and the conclusions from studies have often been contradictory in trying to rationalize torsional barrier heights *via* hyperconjugation and steric effects.^33^ In some part, this arises from the different conditions employed in experiments,^30^ but also the difficulty in resolving and assigning the structure seen in the spectra; additionally, vibration-torsional coupling has been shown to affect deduced barrier heights from spectra^17^,^34^,^35^ and so sometimes incorrect values have been considered and unreliable conclusions therefore reached. It is clear that the introduction of the methyl group increases the density of states (DOS), but of course this only makes a difference if there are mechanisms by which to couple to these.

**Fig. 1:**
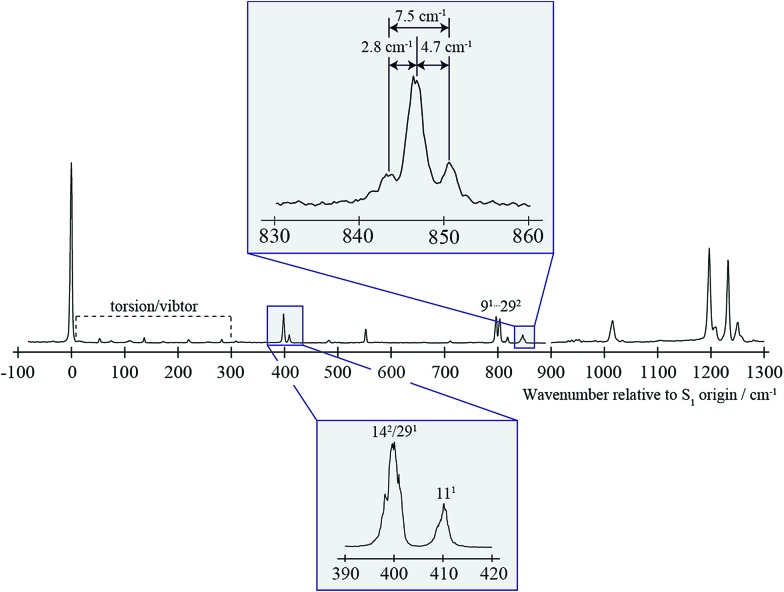
0–1300 cm^–1^ region of the REMPI spectrum of *p*FT. Above is shown an expanded view of the ∼847 cm^–1^ band, that is the subject of the present study. Below is shown an expanded view of the bands close to 400 cm^–1^ which are discussed in the text and whose assignment was discussed in detail in ref. 38. The torsion/vibtor region indicated was also discussed in ref. 38, while the 9^1^/29^2^ bands were discussed in ref. 39.

In recent work, we have employed the technique of zero-electron-kinetic-energy (ZEKE) spectroscopy to study coupling in the one-rotor systems, toluene^36^,^37^ and *p*FT,^38^,^39^ as well as the two-rotor system, *p*-xylene.^40^,^41^ In the present work, we focus on a small group of transitions of *p*FT located in the S_1_ state in a relative wavenumber range 835–855 cm^–1^. These features have been studied relatively recently by Davies and Reid^33^ using time-resolved photoelectron spectroscopy. We shall reassign the main transition as well as the satellites (see Fig. 1) on the basis of the activity seen in the ZEKE spectra together with the results of our previous work on *p*FT.^38^,^39^ The present assignments are confirmed by two-dimensional laser-induced fluorescence (2D-LIF) spectroscopy, which differ from those of earlier studies.^33^,^42^,^43^ We shall make reference to vibration-torsion (vibtor) levels, which occur when torsional levels of the methyl rotor interact with ring-localized vibrations. We shall demonstrate that the main carrier of the transition strength is an overtone of an out-of-plane vibration, and that the primary coupling occurs from one particular torsional level of this overtone level to particular vibtor levels of various combination bands. This involves different torsional levels that facilitate coupling between vibrations of different symmetry. In showing this, we shall demonstrate that it is the torsion-induced change in symmetry requirements that is a central driver of this coupling, but aided also by the increase in the DOS *via* the torsional levels of the methyl group, each of which can combine with the various vibrational energy levels *via* vibration-torsional coupling.

The technique of two-dimensional laser-induced fluorescence (2D-LIF)^44^ is a combination of the standard laser-induced fluorescence (LIF) and dispersed fluorescence (DF) techniques. It has been used to analyze mixtures by Neij *et al.*^45^ and Kable and coworkers,^46^ and also as a detailed probe of rovibronic structure.^44^,^47^,^48^ More recently, it has given highly informative information on the role of internal rotation in vibrational energy dispersal by Lawrance, Gascooke and coworkers.^14^,^17^,^23^,^34^,^44^ Although DF and LIF spectra can be recorded separately, each can be obtained from a 2D-LIF spectrum; and indeed, there is much more information in a 2D-LIF spectrum than in the separate cases.^44^

## 2. Experimental

The apparatus used for the resonance-enhanced multiphoton ionization and ZEKE experiments has been described previously in detail elsewhere.^30^ Briefly, the vapour above room temperature *para*-fluorotoluene (99% purity, Alfa Aesar) was seeded in ∼1.5 bar of Ar and the gaseous mixture passed through a general valve pulsed nozzle (750 μm, 10 Hz, opening time of 180–210 μs) to create a free jet expansion. The focused, frequency-doubled outputs of the two dye lasers were overlapped spatially and temporally and passed through a vacuum chamber coaxially and counterpropagating. Here, they intersected the free jet expansion between two biased electrical grids located in the extraction region of a time-of-flight mass spectrometer, which was employed in the REMPI experiments. These grids were also used in the ZEKE experiments by application of pulsed voltages, giving typical fields (*F*) of ∼10 V cm^–1^, after a delay of up to 2 μs, where this delay was minimized while avoiding the introduction of excess noise from the prompt electron signal. The resulting ZEKE bands had widths of ∼5–7 cm^–1^.

The excitation laser was a dye laser (Sirah Cobra-Stretch) operating with C540A and pumped with the third harmonic (355 nm) of a Surelite III Nd:YAG laser. The ionization laser was a dye laser (Sirah Cobra-Stretch) operating with Pyrromethene 597, pumped with the second harmonic (532 nm) of a Surelite I Nd:YAG laser. The fundamental outputs produced by each dye laser were frequency doubled using BBO and KDP crystals for the pump and probe lasers, respectively.

The apparatus used for recording the 2D-LIF spectra has only recently been constructed and so is now described. The free jet expansion of *p*FT in Ar was produced in the same way as for the REMPI and ZEKE experiments, albeit in a separate chamber. The third harmonic of a Nd:YAG laser (Surelite III) was used to pump a dye laser (Sirah Cobra-Stretch) operating with C503, the frequency-doubled output of which intersected the free-jet expansion at *X*/*D* ∼20. Perpendicular to the excitation laser beam, the resulting fluorescence is collimated and focused either onto a photomultiplier tube (Hamamatsu, H10721-01), allowing a LIF spectrum to be recorded, or onto the entrance slits (200 μm) of a 1.5 m Czerny Turner spectrometer (Sciencetech 9150) operating in single-pass mode, dispersed by a 3600 groove per mm grating, and then collected by a CCD camera (Andor iStar DH334T). With this set-up, approximately 300 cm^–1^ of the dispersed fluorescence may be imaged across the CCD at the wavelengths investigated herein.

At a fixed grating angle of the spectrometer, the wavenumber of the excitation laser was scanned, and at each excitation wavenumber the image was accumulated for ∼5000 laser shots. This produced a 3D surface of intensity as a function of the excitation and dispersed fluorescence wavenumber, denoted a 2D-LIF spectrum.^44^ In the spectral regions scanned here, scattered laser light was not an issue, and so the camera was gated ∼10 ns before the laser fired, and the duration of the gate was 300 ns.

The laser was calibrated using the well documented I_2_ absorption spectrum by recording a LIF spectrum of iodine held in a room temperature static cell. Following laser calibration, scattered laser light at differing wavenumbers was passed through the spectrometer and then detected by the camera, while the grating angle of the spectrometer was fixed, allowing calibration of the fluorescence window of interest. We determine an error in absolute and relative excitation wavenumber to be ≤1 cm^–1^ and ≤2 cm^–1^, respectively, for dispersed fluorescence, with the uncertainties determined by the resolution of the laser, band widths and, in the case of the 2D-LIF spectra, the resolution of the image from the CCD camera system.

## 3. Results

### Level labels

The neutral ground state and first electronically excited states are labelled S_0_ and S_1_ in the usual way, with the ground state cation labelled D_0_^+^. We label vibrations in terms of the *D*_*i*_ labels for *para*-disubstituted benzenes, discussed in ref. 49, and torsional levels by the torsional quantum number *m*, with vibtor levels being a combination of these. Treating the methyl group as a point mass gives the point group symmetry of *p*FT as *C*_2v_ and the phenyl-ring-localized vibrations may be viewed as belonging to one of the four *C*_2v_ symmetry classes. However, when considering torsions and vibtor levels, we are required to use the molecular symmetry group,^50^,^51^ which is *G*_12_ for *p*FT. We shall label transitions with a number or *m* to identify the vibration or torsion, respectively, with upper and lower state quantum numbers given by super- and subscripts, in the usual way (we shall often omit the starting quantum numbers of a transition, as these will be clear from the context). Note that the *m* levels are integral, and usually come in degenerate positive and negative number pairs for non-zero *m* (although conventionally only the positive number is given in specifying transitions). The exceptions are those levels that correspond to non-zero multiples of three, which are split in a hindered rotor system. In the present case, the only two pertinent pairs are *m* = 3(+) and 3(–), and 6(+) and 6(–), which are each formed from linear combinations of the corresponding *m* levels that are degenerate in the free rotor. The *G*_12_ symmetries of the *m* levels, and the corresponding ones for the vibrations, are given in Table 1. The symmetries of levels corresponding to vibrational combinations and overtones, as well as vibtor level symmetries, can be found in the *G*_12_ molecular symmetry group using a direct product table for the *D*_3h_ point group, to which it is isomorphic.

**Table 1:** Symmetries of vibrations and torsions in the *G*_12_ molecular symmetry group

*C*_2v_ Vibration^*a*^	Torsional level, *m*	*G*_12_ Symmetry
*a*_1_	0, 6(+)	*a*_1_′
*a*_2_	6(–)	*a*_2_′
*b*_2_	3(+)	*a*_1_′′
*b*_1_	3(–)	*a*_2_′′
	2, 4	*e*′
	1, 5	*e*′′

^*a*^These are the symmetry classes in the *C*_2v_ point group that results if the methyl group of *p*FT is treated as a point mass; the corresponding molecular symmetry group classes are given in the final column.

### Nuclear spin

Of great importance is that nuclear spin means that the lowest two torsional levels in *p*FT cannot interconvert in our free jet expansion (see ref. 40 for a more-detailed discussion of nuclear spin effects in both *p*FT and *para*-xylene). As such, both the *m* = 0 and *m* = 1 levels will be populated, even under the supersonic-jet-cooled conditions employed here.

As discussed in ref. 14, 17 and 40, and in earlier work cited therein, transitions involving Δ*m* = 0 are expected to be the most intense. Further, since the internal rotational constant is not expected to change substantially between electronic states by analogy with toluene,^17^ transitions involving both sets of *m* = 0 and 1 vibtor levels are expected to be overlapped for each vibrational transition, including the origin – in the absence of any significant perturbation (see below); generally, we shall simply label these overlapped transitions with the vibrational label. In addition, Δ*m* = 3 changes are also expected to be observable in a number of cases, induced by a generalized Herzberg–Teller (HT) vibronic interaction, with Δ*m* = 6 transitions expected to be very weak, and any higher changes not observable.^23^,^40^,^52^

### Symmetry and spectral activity

The assignment of the various spectra is underpinned by the activity we expect to see in the spectra based on symmetry, and so we summarize that here. We shall employ the *G*_12_ molecular symmetry group labels, which are given in Table 1.

First, we consider the transitions arising from the S_1_ ← S_0_ excitation. We note that, under the jet-cooled conditions employed, we are exciting from the zero-point vibrational level of the vibrations in the S_0_ state, which has *a*_1_′ symmetry. This means we expect to see Franck–Condon (FC) allowed vibrations of overall *a*_1_′ symmetry. We also expect to see *a*_1_′′ symmetry vibrations as a result of Herzberg–Teller (HT) coupling, which is a vibronic coupling, “intensity stealing”, mechanism; less appreciated is that fact that this mechanism can also affect the relative intensities of *a*_1_′ symmetry bands.^53^

From a Franck–Condon point of view, the above considerations also hold during the D_0_^+^ ← S_1_ ionization step, *i.e.* with regard to expected activity in the ZEKE spectra. There are, however, two important differences: first we will ionize from a selected S_1_ level, which will have a particular symmetry (rather than just being the *v* = 0 level as for the S_1_ ← S_0_ transition); and secondly, we have found in earlier work that we see activity in ZEKE spectra to low-wavenumber from levels that have the same symmetry as the intermediate level, or that arise from *a*_2_′′ and *a*_2_′ symmetry changes, arising from levels that are components of a selected vibrational or vibtor combination band. The latter are thought to arise from HT interactions in the cation. Because of the high propensity for Δ*v* = 0 transitions, the assignment of the most intense band in a particular ZEKE spectrum will generally correspond to that of the excited intermediate level; other significant bands are expected to be of the same symmetry as the intermediate level, with HT-induced bands also being expected in some cases.

In the 2D-LIF spectra, corresponding to the S_1_ ↔ S_0_ transition, we expect to see similar activity as in the REMPI spectra of the S_1_ ← S_0_ transition, but noting that again we commence at a selected intermediate level, with a particular symmetry.

### Vibration-torsional coupling

The above discussion assumes the absence of any coupling between the torsional, vibrational or vibtor levels. However, it transpires that such coupling pervades the spectra of molecules that contain one or more internal rotors.^14^,^17^,^38^,^40^ It has been suggested that the role of the *m* = 1 levels could serve to increase the range of coupling in such molecules^14^ and we concur with this, and indeed will demonstrate this to be the case in the present work. First, we note that in the S_1_ ← S_0_ transition, the FC-active vibrations will be of *a*_1_′ symmetry and so corresponding vibtor levels with *m* = 0 will also be of *a*_1_′ symmetry. In these cases, corresponding selection rules (for both FC and HT transitions) apply to the vibrational levels as when considering point group symmetry. We also expect to see transitions involving totally symmetric (*a*_1_′) vibtor levels such as 20^1^*m*^3(–)^ and 19^1^*m*^3(–)^;^17^,^38^ these will appear more weakly, but clearly will provide extra activity alongside the pure vibrational (*m* = 0) transitions – such transitions have been seen in toluene^17^ and *p*FT.^38^ For S_1_ → S_0_ and D_0_^+^ ← S_1_ transitions, the initial level will strongly affect the observed activity, with Δ*v* = 0 and Δ*m* = 0, abbreviated to Δ(*v*,*m*) = 0, transitions expected to be the most intense, but accompanied by other transitions to levels of the same symmetry as the initial level, together with weaker Δ*m* = 3 transitions.

We now move on to consider the *m* = 1 (*e*′′ symmetry) levels, which are also populated in our experiment, as noted above. If we now consider vibtor levels that involve *a*_1_′ vibrations and *m* = 1, then their transition wavenumbers will generally be essentially coincident with the corresponding transitions involving the *m* = 0 level, owing to the expected similarity of the internal rotational constant in the two electronic states. This situation may change as a result of coupling between vibtor levels, as we shall see. Reference to a direct product table for the *D*_3h_ point group (recall, this is isomorphic to *G*_12_) shows that the *m* = 1 levels of both *a*_1_′ and *a*_2_′ vibrational levels can interact (both *e*′′ overall), and the same is true for *a*_1_′′ and *a*_2_′′ vibrational levels (both *e*′ overall). Hence, the presence of *m* = 1 population, as a result of nuclear spin, widens the possibilities for coupling between vibrations of different symmetry. However, there is a further “loosening” of the selection rules, because vibtor levels that arise from vibrational levels of *a*_1_′′ and *a*_2_′′ symmetry and *m* = 2 (*e*′′ symmetry overall) can couple to vibtor levels that arise from vibrational symmetries *a*_1_′ and *a*_2_′ and *m* = 1 (also *e*′′ symmetry overall). Lastly, a combination of the HT mechanism and vibration-torsion coupling can allow coupling between levels of *e*′ and *e*′′ symmetry, providing a means for coupling between vibrational levels of all four symmetry classes.

### Overview of spectra

In Fig. 1 we show the first 1300 cm^–1^ of the REMPI spectrum of the S_1_ ← S_0_ transition of *p*FT. The low-wavenumber region, including transitions involving “pure” torsional levels and some vibtor levels, has been discussed in depth previously.^38^ This region also includes a set of close-lying states at ∼400 cm^–1^, whose assignment to, at most weakly, interacting levels was also discussed in that work, and which will be seen to be pertinent to the present study. To higher wavenumber, as well as a number of weak features, the spectrum is dominated by sets of transitions, many of which involve the overtones and combinations of the states that contribute to the ∼400 cm^–1^ region. In the present work, we shall focus on the feature that appears at ∼847 cm^–1^ and for which an expanded version is shown in Fig. 1.

**Fig. 2:**
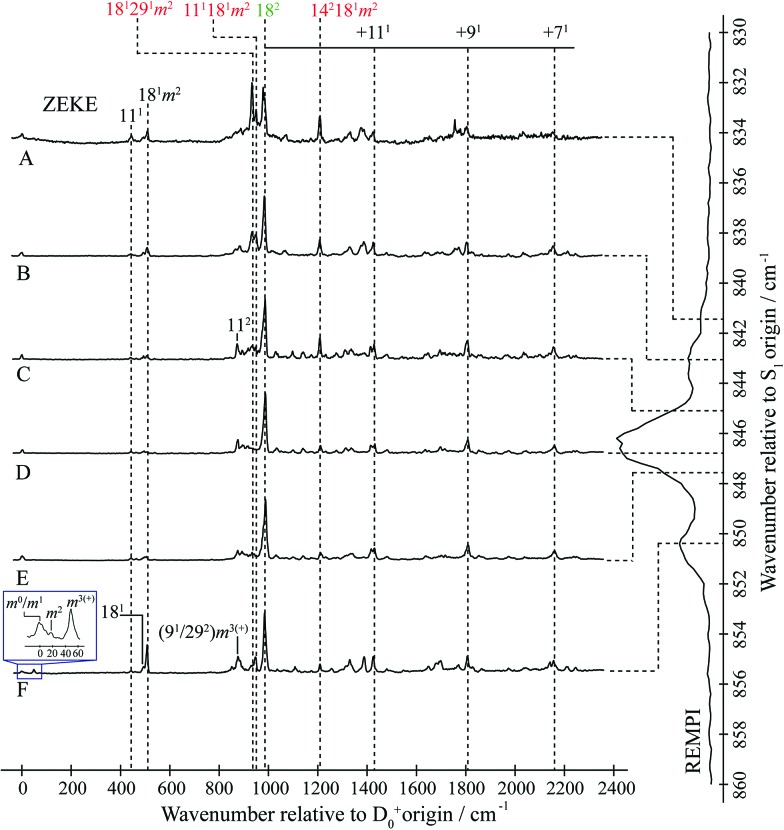
“2D-ZEKE” spectrum of *p*FT recorded at the excitation positions indicated, with the assignments given for the main features. In addition, we indicate the locations of repeated structure associated with combinations of the main bands with totally-symmetric vibrations. The vertical trace on the right-hand side shows the relevant section of the REMPI spectrum (see Fig. 1), with dashed lines indicating the excitation positions at which the ZEKE spectra were recorded. When exciting *via* position F we see clear evidence of involvement of the *m* = 1 level *via* the *m*^2^ band in the expanded trace The presence of the *m*^3(+)^ band is consistent with activity from the *D*_9_*m* = 3(+) and 2*D*_29_*m* = 3(+) levels on the high wavenumber side of the REMPI feature. See text for further details.

In Fig. 2 we show a set of ZEKE spectra recorded when exciting at different wavenumbers corresponding to positions within the ∼847 cm^–1^ band profile, with these positions indicated. Taken together, these constitute what we term a two-dimensional ZEKE (2D-ZEKE) spectrum, which will be seen to be analogous to the 2D-LIF spectra presented later, albeit less comprehensive. It can be seen that each ZEKE spectrum has a contribution from an intense band at 988 cm^–1^; additionally, there are many other bands that occur in the spectra, some of whose relative intensities are markedly different as the sequence is traversed. This differing activity is a reflection of the varying nature of the S_1_ levels accessed across the ∼847 cm^–1^ feature. Of note are other significant bands at 509 cm^–1^, 933 cm^–1^, 950 cm^–1^ and 1209 cm^–1^, with various other bands in the ranges 850–955 cm^–1^ and 1000–1200 cm^–1^. To higher wavenumber, we also see repeats of the main structure that lies within the 850–1220 cm^–1^ range, which are assigned to the same transitions, but with each upper level being in combination with the *D*_11_, *D*_9_ and *D*_7_ totally symmetric vibrations, as indicated in Fig. 2. As a consequence, in the discussion below we shall focus on the 509 cm^–1^ band, and key features in the Δ(*v*,*m*) = 0 region (850–1220 cm^–1^). These regions are also the primary focus of the 2D-LIF spectra reported below.

### Assignment of ZEKE spectra

In ref. 42, the main band at 847 cm^–1^ was assigned to the 18a^1^ transition (employing Wilson^54^/Varsányi^55^ notation). We have noted in ref. 49 and 56 that really this mode should be labelled *ν*_19a_ and would then correspond to mode *D*_8_ in the nomenclature used here. This assignment, perpetuated in our previous study of *p*FT using ZEKE spectroscopy^43^ and in the work of Davies and Reid,^33^ appears to have arisen because of the close agreement of the observed Δ*v* = 0 1000 cm^–1^ DF band with the expected S_0_ value for “*ν*_18a_”. Given the good agreement between the calculated wavenumbers of the other *a*_1_ modes with the experimental values in the S_1_ state (see Table 2), it seems unlikely that this assignment is correct. In the present work, we therefore sought an alternative assignment, and quickly identified the overtone transition 18^2^ as the most likely, fitting the REMPI feature, the main ZEKE band at 988 cm^–1^, and the main DF band at 1000 cm^–1^. The reassignment here thus gives good agreement between calculated and experimental values for all three electronic states considered.

**Table 2:** Calculated and available experimental wavenumbers for vibrations relevant to the present work

Vibration	S_0_		S_1_		D_0_^+^	
	Experimental^*a*^	Calculated^*b*^	Experimental^*c*^	Calculated^*c*^	Experimental^*c*^	Calculated^*c*^
*D*_7_	1157	1145		1120	1170	1158
*D*_8_		1005		954		969
*D*_9_	843	827	797	805	824	811
*D*_11_	453	446	408	410	440	437
*D*_14_	(411)	418	199	172	350	356
*D*_18_	(499)	500	426	468	494^*d*^	488
*D*_19_	(335)	330	242	243	271	266
*D*_20_	(144)	141	110	110	111	109
*D*_29_	426	414	399	395	416	412
*D*_30_	(308)	298	311	307	320	313

^*a*^ Ref. 49 and present work. Values in parentheses are determined from first overtone bands observed in the present work.

^*b*^See ref. 49. B3LYP/aug-cc-pVTZ, scaled by 0.97.

^*c*^See ref. 38 and 39. (TD-)B3LYP/aug-cc-pVTZ, scaled by 0.97. Note that calculated wavenumbers for the S_1_ state vibrations of *a*_2_ and *b*_1_ symmetry are not as reliable as those of the *a*_1_ and *b*_2_ symmetry ones; to date vibrational wavenumbers for the S_0_ and D_0_^+^ states have proven to be reliable across all four symmetry classes at this level of quantum chemistry.

^*d*^Refined from that presented in ref. 38 and 39.

The ZEKE spectra (see Fig. 2) recorded *via* the satellite REMPI bands each also show the main 18^2^ band, which is (generally) the most intense, but other notable activity is evident. Of great interest was the ZEKE band at 509 cm^–1^ – being so low in wavenumber, there are very few assignments for this band, but its origin was problematic for some considerable time. Equally puzzling was the ZEKE band at 1209 cm^–1^. After eliminating the possibility of various pure vibrational bands, by comparing the ZEKE and DF activity, vibtor assignments were considered. An assignment evolved that fitted all of the key bands in both the ZEKE and DF spectra that, remarkably, consists of transitions that may be viewed as combinations of the *D*_11_, 2*D*_14_ and *D*_29_ vibrations, each with the *D*_18_*m* = 2 vibtor level. That is, the transitions corresponding to the satellite bands of the ∼847 cm^–1^ feature are analogues of the main bands that give rise to the bands at ∼400 cm^–1^ (see ref. 38, Fig. 1 and comments below). Note that the 14^1^*m*^6(–)^ transition was also assigned in our previous study,^38^ but as the terminating level is already a vibtor level it cannot form combinations with another vibtor level.

This assignment is consistent with the 1209 cm^–1^ ZEKE band being 14^2^18^1^*m*^2^ and the 509 cm^–1^ band being a symmetry-allowed “component” band (see earlier comments), 18^1^*m*^2^. The latter band is most prominent when exciting *via* the two satellite bands, and is significantly weaker when exciting *via* the main central band. In addition, the relative intensity of the 509 cm^–1^ and 1209 cm^–1^ ZEKE bands is not consistent across the spectra. The explanation for this will be seen to be that the 509 cm^–1^ band, assigned to 18^1^*m*^2^, arises from a number of transitions in this region that commence from vibtor levels containing *D*_18_*m* = 2. We also note the presence of the 18^1^ ZEKE band in many of the spectra, a vibronically-allowed “component” band, and also the *m*^2^ band that can sometimes be discerned as a weak shoulder on the origin band – see the insert in Fig. 2, trace F.

By analogy with the 400 cm^–1^ spectral regions of the REMPI spectrum (see Fig. 1 and ref. 38), we would expect the 11^1^18^1^*m*^2^ and 18^1^29^1^*m*^2^ ZEKE bands at around 950 cm^–1^ and 930 cm^–1^ and so we assign the 950 cm^–1^ and 933 cm^–1^ ZEKE bands to these transitions – see Fig. 2. The coupling mechanism will be discussed below.

We note that we see bands assigned as 9^1^*m*^3(+)^ and 29^2^*m*^3(+)^ when exciting the upper satellite (position F) of the 847 cm^–1^ band; these are analogues of the main bands that appear at ∼800 cm^–1^ that are labelled in Fig. 1, which were discussed in depth in ref. 39. Also in this spectrum (see insert) is the associated symmetry-allowed *m*^3(+)^ “component” band. Note that there are many other features in the spectrum, but we refrain from a detailed discussion of those in the present work.

In summary, we are able to rationalize the main activity in the ZEKE spectra in terms of combinations of the *D*_18_*m* = 2 vibtor level with each of the three vibrations that have wavenumbers close to 400 cm^–1^, studied in our earlier work.^38^ We emphasise that these vibtor combinations would not be expected to have any appreciable intensity without interacting with an optically bright state (see below). We now move on to discuss the fluorescence spectra.

**Fig. 3:**
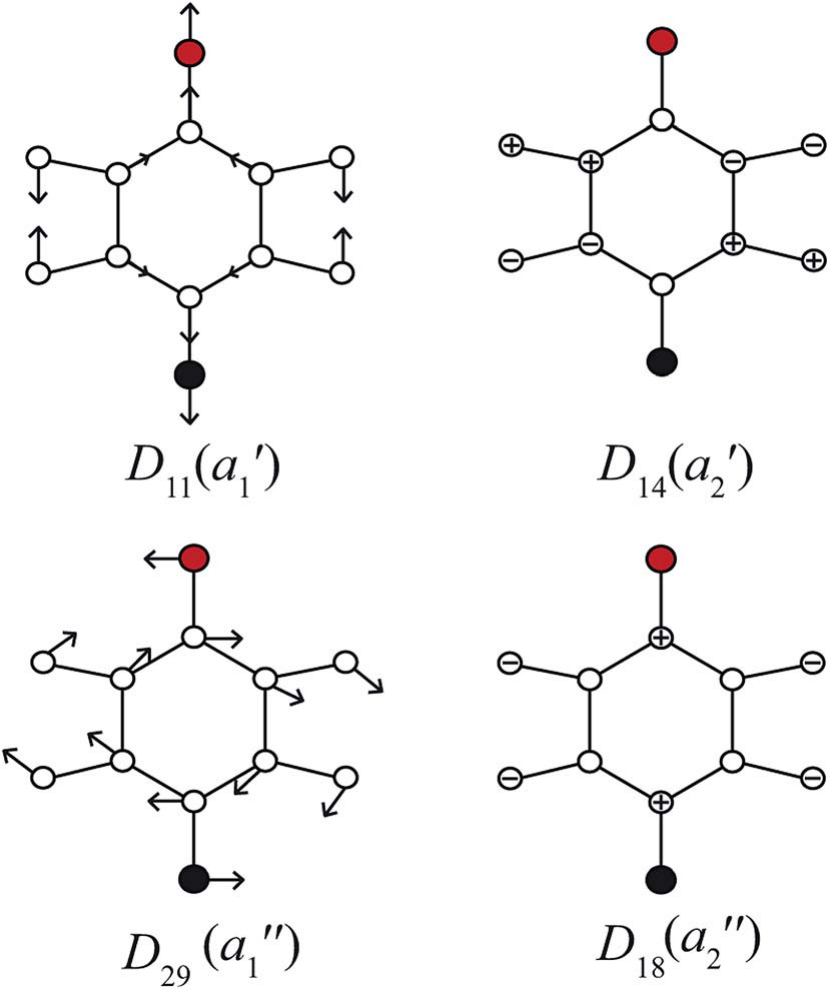
Schematics for the four vibrational modes of main interest to the assignment of the feature at ∼847 cm^–1^ – see text. The methyl group is represented as a point mass (red), with the F atom being black – these modes are essentially identical to those for *para*-difluorobenzene in ref. 49.

In Fig. 3, we show the mode diagrams of the key vibrations that make up the assignments of the feature under discussion. These may be seen to involve the two in-plane vibrations, *D*_11_ and *D*_29_, and the out-of-plane vibrations, *D*_14_ and *D*_18_.

### 2D-LIF and coupling

**Fig. 4:**
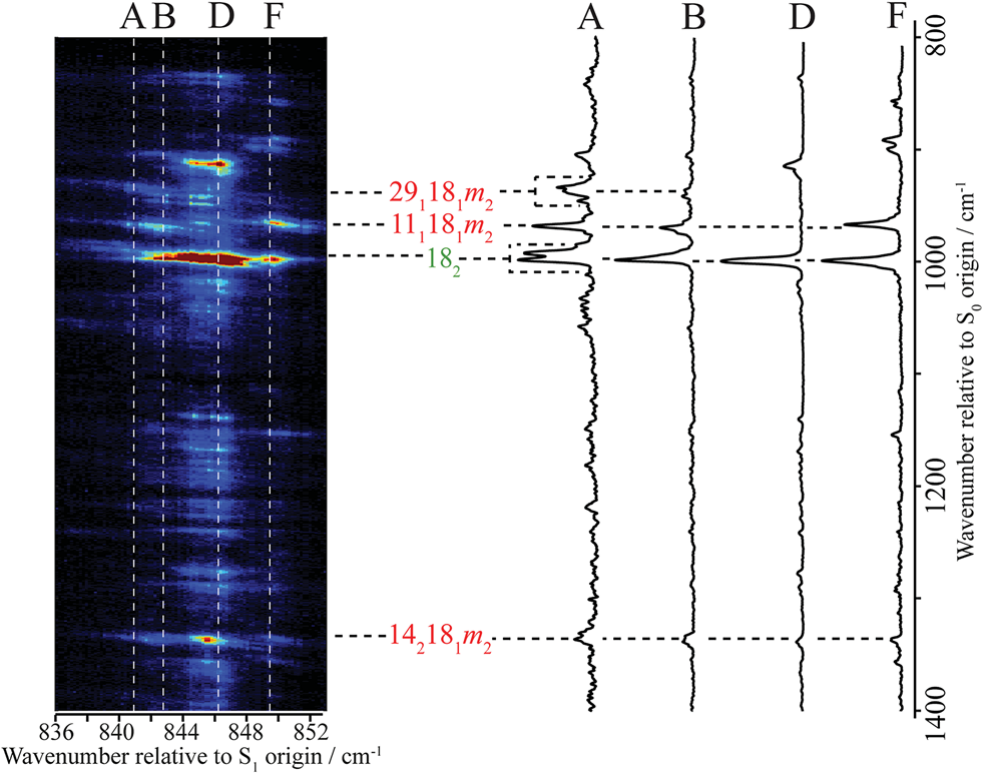
A section of the 2D-LIF spectrum of *p*FT corresponding to the Δ(*v*,*m*) = 0 region is shown on the left-hand side. The intensity is indicated by colouring on a linear scale, with red being the most intense, moving through light blue to dark blue for the least intense features; black indicates zero intensity. The transitions showing the main structure are indicated and these are linked to DF spectra obtained by slicing vertically through the 2D-LIF image at the indicated positions, with the letters referring to the expanded REMPI trace in Fig. 2. Note that the relatively high resolution here, together with the form of the rotational structure associated with each spectral feature in the 2D-image, means that caution is merited when considering apparent double bands close in wavenumber – see text.

**Fig. 5:**
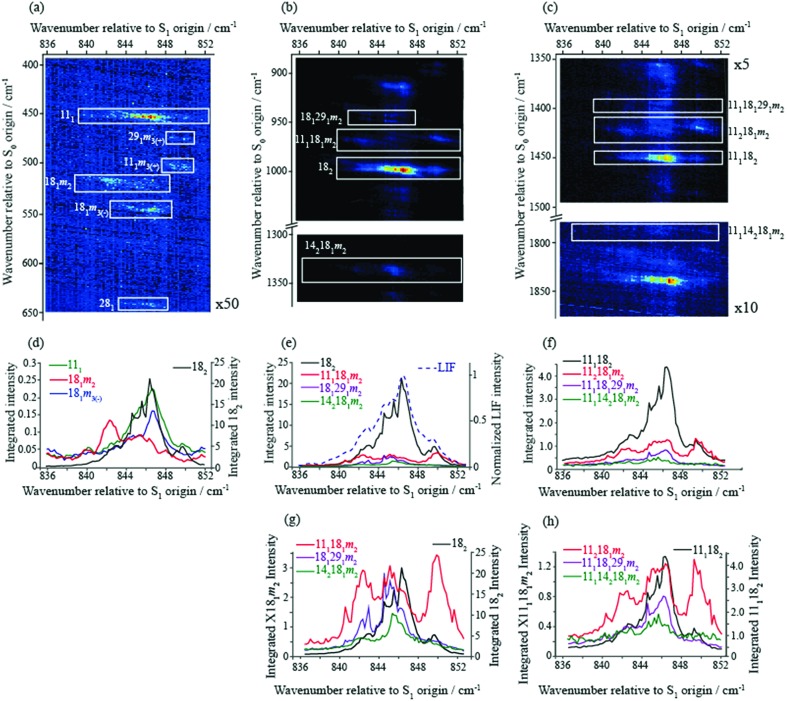
(a) Section of the 2D-LIF spectrum, covering a wavenumber region below the Δ(*v*,*m*) = 0 region, where the main activity corresponding to the transitions that are the focus of attention in the main text are indicated. (b) Section of the 2D-LIF spectrum, covering the Δ(*v*,*m*) = 0 region, where the main activity corresponding to the transitions that are the focus of attention in the main text are indicated. (c) Section of the 2D-LIF spectrum covering the Δ(*v*,*m*) = 0 region plus *D*_11_, where the main activity corresponding to the transitions that are the focus of attention in the main text are indicated. (d)–(f) Show “partial” LIF spectra obtained by vertical integration across horizontal slices of the 2D-LIF image, each corresponding to each of the key transitions in the 836–852 cm^–1^ range, as indicated. The dashed line in (e) shows a conventional LIF spectrum and may be seen to have a similar structure to the 18_2_ integrated trace, and is also similar to the REMPI spectrum in Fig. 1. In (g) and (h) we have scaled the contributions for easier comparison. Note that the combination vibtor traces do not have a maximum intensity at the expected positions, owing to interactions with the bath states (see text). Also note that the integrated traces in (h) are likely affected by overlapping features. The position of the legends, and the axis labels, indicate which scale refers to which trace.

In the present work, we have recorded 2D-LIF spectra across the main ∼847 cm^–1^ feature, when collecting the fluorescence across wavenumber regions expected for the Δ(*v*,*m*) = 0 bands for the levels discussed above (see Fig. 4 and 5). In addition, we also report the corresponding spectra in the higher-wavenumber region that corresponds to the same transitions, but involving combinations with the *D*_11_ vibration (see Fig. 5). These spectra show the changing activity across the features in detail, and also indicate the couplings between them. In principle, these show more-complete data than the ZEKE spectra that are shown in Fig. 2, but *via* transitions to a different electronic state, and so the spectra are complementary. Vertical slices through the 2D images give a section of the DF spectrum at that excitation wavenumber, while complete vertical integration will give an LIF spectrum from the range of fluorescence collected.^44^ Although there is a lot of information contained in the 2D-LIF images, for the purposes of the present paper we focus on the main activity and couplings between the levels assigned in the ZEKE spectra, discussed above. We make use of the fact that we can integrate across a horizontal slice of a 2D-LIF spectrum that corresponds to accessing a particular level in the ground state (the vertical breadth of a feature is due to underlying rotational structure). Such integrations will give an indication of the activity of a particular S_0_ vibration/vibtor level across the range of excitation. We can also just take a single horizontal slice across a spectrum, which will give localized information on the activity – see below.

A section of the 2D-LIF image showing the main Δ(*v*,*m*) = 0 regions is shown in Fig. 4. If the above assignments from the ZEKE spectra are correct, then corresponding bands should appear in the DF spectra, when exciting at similar wavenumbers. On the right-hand side of Fig. 4 are sections of the DF spectrum obtained by taking vertical slices through the 2D image at the positions indicated; these correspond to positions in the REMPI spectrum, as indicated by the letters – see expanded trace in Fig. 2. We note that a strong band at 1000 cm^–1^ appears across the set of DF spectra that is analogous to the 988 cm^–1^ ZEKE band, and so assigned to the 18_2_ transition.

Of note is that the appearance of a feature in a DF spectrum can depend on the wavenumber excited, as is evident from the 2D-LIF spectra. This is because the resolution here is sufficient to pick out different portions of the rotational profile. This occurs because of the overall rotational structure of a 2D-LIF band, which generally occurs as variations on a “star” or “cross”, depending on the transition polarization type^44^ (similar comments were made regarding the differing ZEKE profiles seen when exciting through different tranches of the rotational profile^38^). From the expanded views in Fig. 5, it can be seen that a vertical cut through the centre of a cross would give a single band in the DF spectrum, while a cut to slightly higher or lower wavenumber could pick up the “arms” and so lead to a double feature. Thus, caution is required in identifying close-lying vibrational bands in these higher-resolution DF spectra, to ensure that the bands do not simply arise from the same transition, and the 2D-LIF image is key in identifying this.

As noted above, the 18_2_ band appears in all of these DF spectra, with its wavenumber in close agreement both with previous IR and Raman studies and also with the calculated values (Table 2). When exciting *via* the positions A, D and F, we see a strong band at 966 cm^–1^ – see Fig. 4 – assigned to 11_1_18_1_*m*_2_, and this confirms a major contribution from *D*_11_*D*_18_*m* = 2 across this excitation wavenumber range; this band is notably absent in the DF spectrum when exciting at position D. Present throughout, but most prominent when exciting at position B, we see a band at 1339 cm^–1^, which can be assigned to 14_2_18_1_*m*_2_ and a band at 943 cm^–1^ when exciting at position A, which can be assigned as 18_1_29_1_*m*_2_. Thus, we have confirmation that the contributions here are combinations that are analogous to the three main contributions at ∼400 cm^–1^ that were deduced from the ZEKE spectra (see above).

In Fig. 5(a)–(c), we show different regions of the 2D-LIF spectrum, with the pertinent transitions indicated. The spectrum in Fig. 5(b) is the Δ(*v*,*m*) = 0 region, while Fig. 5(a) shows the lower-wavenumber region, where the 11_1_, 18_2_*m*_2_ and 28_1_ features can be seen; additionally, we see another band in this region assigned as 18_1_*m*_3(–)_, which is of *a*_1_′ symmetry. Note that the spectra in Fig. 5(a) and (c) are each significantly weaker than that in 5(b) indicating the Δ(*v*,*m*) = 0 region is the most intense, as expected.

Directly below the 2D-LIF spectra, are “partial” LIF spectra obtained by the vertical integration of a horizontal slice corresponding to the rotational transitions for a particular feature; the lowest figures show traces where the features have been scaled to allow a more direct comparison. In Fig. 5(e) we also show the LIF spectrum, and this can be seen to have a very similar profile to that obtained from the 18_2_ activity, and this is also the case for the corresponding integrated trace for 11_1_18_2_ in Fig. 5(f). This indicates that the form of the LIF spectrum in this wavenumber range is dominated by transitions involving 2*D*_18_ and its coupling to other nearby levels. Unfortunately, we are not able to disentangle the contributions from 18_2_*m*_1_ and 18_2_*m*_0_ since the rotational profiles overlap; as a consequence, the trace labelled 18_2_ consists of both *m* = 0 and *m* = 1 contributions. The other traces correspond to the transitions terminating in the other main states under consideration: 11_1_18_1_*m*_2_, 14_2_18_1_*m*_2_ and 18_1_29_1_*m*_2_. As may be seen from the plots, all three demonstrate activity across the wavenumber range. Of note is that the activity in the centre of the spectrum is localized on the low-wavenumber side of the main 18_2_ band, which we conclude is the *m* = 1 contribution (see below). This is also confirmed by the peak in the 18_1_*m*_3(–)_ integrated trace – see Fig. 5(d) – being coincident with the peak in the 18_2_ integrated trace that is to higher wavenumber, and corresponds to the 2*D*_18_*m* = 0 level. Note that the S_0_*D*_18_*m* = 3(–) level (*a*_1_′ symmetry) is not accessible (symmetry forbidden) from the S_1_ 2*D*_18_*m* = 1 level (*e*′′ symmetry), but is accessible from 2*D*_18_*m* = 0. The separation of the *m* = 0 and *m* = 1 components of the 18_2_ band must be as a result of various vibration-torsional interactions, which are discussed below. The fact that each trace shows activity across the spectral range suggests that the 2*D*_18_*m* = 1 level is interacting with each of the three aforementioned vibtor levels, and this will now be discussed further.

The integrated profiles for the 14_2_18_1_*m*_2_, 18_1_29_1_*m*_2_ and 11_1_18_1_*m*_2_ transitions, see Fig. 5(e)–(g), show a rather unexpected behaviour in that there is no dominant band corresponding to the Δ(*v*,*m*) = 0 transition in each case, with the 18_2_ feature always the most intense. Further, the two lowest-wavenumber transitions demonstrate a clear maximum at the position of 18^2^*m*^1^, while the 11_1_18_1_*m*_2_ transition shows approximately equal intensities at the three wavenumbers that correspond to the main REMPI/LIF features. The integrated traces give insight into the coupling mechanisms between each of these levels and will be discussed further in the following section. We also note that in Fig. 5(a) and (d) the 18_1_*m*_2_ transition has a maximum at the position of the 14^2^18^1^*m*^2^ band and extends across the position of 18_1_*m*_1_; on the other hand, there is apparently very little intensity at the expected position of 11^1^18^1^*m*^2^; we rationalize this in terms of Franck–Condon activity for this feature, since both transitions would be symmetry allowed. In addition, there is little intensity in the region of 18_2_*m*_0_, as expected, as this transition would be symmetry forbidden. The intensity profile for the 11_1_ feature appears to follow the 18_2_ profile, suggesting contributions from both *m* levels, and so simply arises from symmetry-allowed activity.

We mentioned earlier that we observed ZEKE bands arising from 9^1^*m*^3(+)^ and 29^2^*m*^3(+)^ when exciting at position F. We also see corresponding features in the 2D-LIF spectrum arising from these levels, confirming their activity. Further, we also see weak 2D-LIF bands whose activity suggests that they also originate from these two levels – these are indicated in Fig. 5(a) and are assigned as 11_1_*m*_3(+)_ and 29_1_*m*_3(+)_. The former band is symmetry allowed while the latter is HT-allowed.

**Fig. 6:**
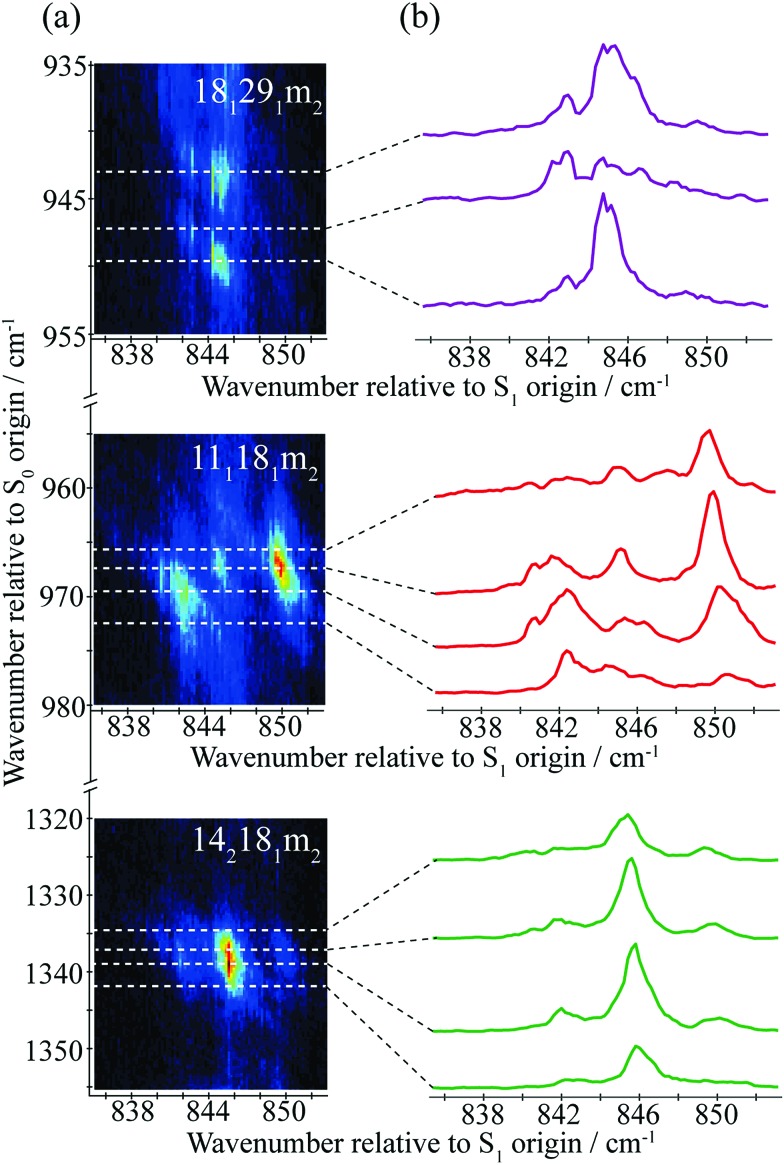
(a) Expanded views of the Δ(*v*,*m*) = 0 features in the 2D-LIF spectra. (b) Traces of the fluorescence intensity accessing the same internal energy of the S_0_ state. These show that the different forms of the rotational profiles can affect the intensity variation across a feature significantly, and the extended forms of some of these can affect the apparent relative intensity of neighbouring features. See text for further comment.

It may be seen from the 2D-LIF images in Fig. 5 and 6 that the band profiles are varied. To gain further insight into the coupling, we also show plots of the variation in the fluorescence activity accessing the same internal energy in the S_0_ state – these are obtained from horizontal cuts across the spectra, and are shown in Fig. 6 for the main Δ(*v*,*m*) = 0 bands. These give a more-detailed insight into the structure of each band, such as the wide extents of the rotational structure for each contributing feature. These also confirm that there is much less intensity in the Δ(*v*,*m*) = 0 regions for each of the 18_1_29_1_*m*_2_ and 14_2_18_1_*m*_2_ bands, with dominance at the positions of 18_2_*m*_1_.

### Vibration-torsional coupling and IVR

To discuss the appearance of the spectra and the coupling mechanism, we first need to cover the language of IVR briefly.^8^ In a frequency-resolved scenario, such as here, we can consider an optically-bright vibronic transition resulting in local activity in a specific region of the excitation spectrum, with the final state termed the zero order bright (ZOB) state. Then, by virtue of proximity in wavenumber, other states of the same symmetry and similar wavenumber can interact with the ZOB state and become mixed with it *via* various coupling mechanisms. This coupling leads to a number of eigenstates having a contribution from the ZOB state and hence appearing in the spectrum, whereas they would be optically “dark” had the coupling not occurred, and so not be seen, and so these are denoted zero-order dark (ZOD) states. If there are only one or two ZOD states coupled, then the situation is termed “restricted” IVR, while if there are a significant number of ZOD states coupled, then this is usually termed statistical or “dissipative” IVR, and the many ZOD states are termed “bath states”. The term zero-order states (ZOSs) is used to refer to ZOB states, ZOD states, or both. In a time-domain experiment, a very short (picosecond) laser pulse coherently excites a set of eigenstates that arise from a particular set of coupled ZOSs, which leads to the formation of a wavepacket. This wavepacket, which consists of a juxtaposition of the eigenstates, will look like the ZOB state at *t* = 0, provided all eigenstates that contain a significant contribution from it are coherently excited. The wavepacket evolves as the phases between the eigenstates changes and at various later times the wavepacket's appearance will be dominated by the ZOD states. If, however, the IVR is dissipative, *i.e.* there many ZOSs coupled, then although the wavepacket will resemble the ZOB state at *t* = 0, the time-dependent signal used to monitor the wavepacket will be seen to decay with time as the initial ZOB state character evolves into the characters of the bath states. There is, however, an interesting intermediate case whereby a small number of ZOD states are strongly coupled with the ZOB state, and also to the bath states; but, the ZOB state itself is only very weakly coupled to the bath states. In such a situation these ZOD states are termed “doorway” states, as they provide an indirect route for the ZOB state to be coupled more strongly to the bath states, which would not occur efficiently were they not there. Although put forward many years ago, the concepts of “doorway” states and the tier model^4^,^8^,^57^ have regained interest *via* the analysis of time-resolved studies. As noted above, the concept of ZOSs can be generalized to include vibrations, torsions, and vibtor levels. (For clarity, we emphasise that although descriptions of time-dependent IVR processes often refer to “population” changes, this is only an apparent change in the population of the ZOSs, while in fact the population of the eigenstates is unchanging. Further, although the vibrational ZOSs are often referred to as “harmonic”, in fact they are always anharmonic in reality (fundamentals, overtones, combinations), with the IVR process providing routes for “off-diagonal” anharmonic coupling between them.)

In the present case, the coupling mechanism for the interactions between the vibtor levels noted above (*D*_11_*D*_18_*m* = 2, 2*D*_14_*D*_18_*m* = 2 and *D*_18_*D*_29_*m* = 2) and the 2*D*_18_ level cannot occur with the *m* = 0 level of the latter (*a*_1_′ symmetry), and in fact involves the 2*D*_18_*m* = 1 level, which is of (*a*_1_′ × *e*′′=) *e*′′ symmetry. The vibtor level *D*_18_*m* = 2 is also of (*a*_2_′′ × *e*′=) *e*′′ symmetry and the same symmetry results when this is in combination with other *a*_1_′ vibrations, such as *D*_11_ and 2*D*_14_. These combination vibtor levels involving *D*_18_*m* = 2 would not be expected to have any significant inherent intensity in the spectrum, and so their appearance only occurs by virtue of the interaction with 2*D*_18_*m* = 1. The activity in the *D*_18_*D*_29_*m* = 2 (overall *e*′ symmetry) vibtor level occurs *via* a generalization of HT vibronic coupling, analogous to the way that the 29^1^ transition is active in the 400 cm^–1^ region.^38^

This picture of the coupling is supported by the 2D-LIF images in Fig. 4 and 5, the integrated plots in Fig. 5, and the slices through the 2D-LIF spectrum in Fig. 6. These clearly show that there is 18_2_ intensity across the whole of the 847 cm^–1^ feature; indeed, the activity of the 18^2^ transition in similar molecules^58^ supports its assignment as the ZOB state. Further, it is clear that there are contributions from each of the three aforementioned vibtor combinations, and so these are ZOD states.

In such a scenario, the intensity of the S_1_ 2*D*_18_*m* = 1 ZOB state character would be “shared out” across the range of excitation wavenumbers, as evidenced in the 18_2_ integrated traces in Fig. 5(d), (e) and (g) while the intensity of the 2*D*_18_*m* = 0 level would be restricted to a relatively localized wavenumber range as is indeed inferred by the 18_1_*m*_3(–)_ integrated intensity trace in Fig. 5(d), which may only be accessed from the S_1_ 2*D*_18_*m* = 0 level (*a*_1_′ symmetry). However, the integrated intensity traces of the 11_1_18_1_*m*_2_, 18_1_29_1_*m*_2_ and 14_2_18_1_*m*_2_ transitions in Fig. 5(e) and (g) are more complicated than one might expect. In a simple vibration-torsion coupling mechanism, five bands would be expected to be observed in the electronic excitation spectrum, representing the four coupled eigenstates:

(i) *D*_11_*D*_18_*m* = 2···2*D*_18_*m* = 1,

(ii) 2*D*_14_*D*_18_*m* = 2···2*D*_18_*m* = 1,

(iii) *D*_18_*D*_29_*m* = 2···2*D*_18_*m* = 1,

(iv) 2*D*_18_*m* = 1···*D*_11_*D*_18_*m* = 2···2*D*_14_*D*_18_*m* = 2···*D*_18_*D*_29_*m* = 2,

(v) the uncoupled 2*D*_18_*m* = 0 level.

In the above, the dominant contribution is given first, and the bands arising from these are likely to be overlapping. DF or ZEKE spectra recorded *via* each of these eigenstates would be expected to be dominated by a transition to the leading term in each case; for example, for the first eigenstate, the 11_1_18_1_*m*_2_ transition would be expected to have the most intense band, with a less intense 18_2_*m*_1_ band also being expected; this is clearly not the case here.

This deviation from expected behaviour may be explained through further couplings involving the ZOSs, the most obvious of which is between the three main ZOD states just discussed. In such a scenario, activity involving all coupled levels would be observed in the ZEKE and DF spectra, with the relative intensities of the bands observed reflecting the composition of the eigenstates. Although the 11_1_18_1_*m*_2_ integrated trace in Fig. 5(e) and (g) shows three peaks, one coincident with the 18_2_*m*_1_ position, and two at the positions of the satellite bands observed in the REMPI spectrum, see Fig. 1 and 2, the approximately equal intensities of each of these peaks is not consistent with such a picture and neither are the behaviours of the 14_2_18_1_*m*_2_ and 18_1_29_1_*m*_2_ traces, each of which show a weak band corresponding to the lower-wavenumber satellite band and a more intense central band, while the higher-wavenumber satellite band is essentially absent.

This indicates that further coupling is present, and this is inferred from the relatively weak, but wide-ranging structure in the ZEKE spectra (see Fig. 2, particularly noting the structure across the range 870–1800 cm^–1^) and in the 2D-LIF spectra (see Fig. 4, particularly noting the wealth of structure in the range 1000–1350 cm^–1^). Both of these suggest that further significant mixing to other ZOSs has occurred, and the *D*_11_*D*_18_*m* = 2, 2*D*_14_*D*_18_*m* = 2 and *D*_18_*D*_29_*m* = 2 levels, rather than being just spectators in the vibrational energy dispersal in this wavenumber range, actually facilitate it; *i.e.* they are acting as doorway states coupling the bright 2*D*_18_*m* = 1 state to a bath of background states. Although some of the weaker bands observed will arise from symmetry-allowed “Franck–Condon” activity from the ZOB state, other structure will be associated with the coupled ZOD states, and there are many such possibilities (see below), which largely arise from vibration-torsional coupling and an effective relaxation in symmetry restraints on coupling between different vibrational levels, which is now discussed.

**Fig. 7:**
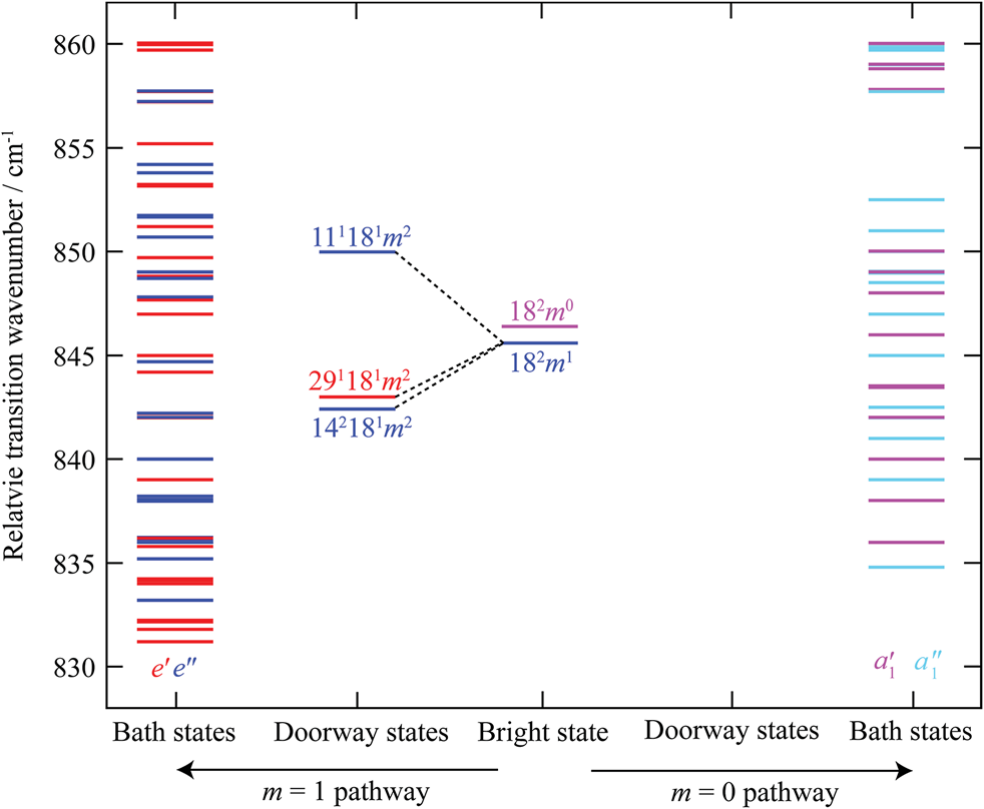
Schematic diagram showing the relative transition wavenumbers of the *m* = 0 and *m* = 1 components of the 2*D*_18_ state, and the combination vibtor levels discussed in the text; each are at the approximate position they appear in the REMPI spectrum (see Fig. 1) (note that the *e*′ and *e*′′ levels including 2*D*_18_*m* = 1, are on an absolute energy scale that is shifted by the *m* = 1–0 spacing in the S_0_ state (∼5.5 cm^–1^) relative to the other levels, including 2*D*_18_*m* = 0). We have labelled these as transitions, rather than as levels. On the far right of the figure are vibrational and vibtor levels that are calculated to have symmetries that allow them to interact directly with 2*D*_18_*m* = 0 (*a*_1_′ symmetry) or *via* a Herzberg–Teller mechanism (*a*_1_′′ symmetry), shown in pink and light blue, respectively. On the far left-hand side are the levels that are calculated to have the correct symmetry to interact directly with the 2*D*_18_*m* = 1 level (*e*′′ symmetry) or *via* a Herzberg–Teller mechanism (*e*′ symmetry), shown in dark blue and red, respectively. Note that there are no doorway states for the 2*D*_18_*m* = 0 level. See text for further discussion.

To illustrate the importance of the relaxation of the symmetry constraints, we have calculated the approximate positions of the various levels in the S_1_ state using vibrational wavenumbers from ref. 39 and the torsional levels from ref. 38. We calculated all of the possible levels from 830–860 cm^–1^, including up to four vibrational quanta and *m* levels up to and including *m* = 6, and indicate their relative wavenumbers in Fig. 7. Excluding the 2*D*_18_ levels, we find that there are only five *a*_1_′ “pure” vibrational combinations that lie in the correct energy range in the S_1_ state that could potentially interact with 2*D*_18_*m* = 0, with another nine vibtor levels of *a*_1_′ symmetry, and *via* HT vibronic coupling, nineteen levels with *a*_1_′′ symmetry; however the coupling to these must be weak/higher order, as these do not have the correct energies to match the most intense bands observed in the spectra. Once we allow additional coupling involving the *m* = 1 state, this opens up twenty five other levels of *e*′′ symmetry and, *via* HT vibronic coupling, thirty levels with *e*′ symmetry. Overall, there are thus 33 levels in the range that could interact with 2*D*_18_*m* = 0, and 55 that could interact with 2*D*_18_*m* = 1. However, evidently many of these interact only weakly, as suggested by the dominance of the bands corresponding to the main doorway states; and indeed, the activity indicates that it is only the 2*D*_18_*m* = 1 level that undergoes sizeable interactions. The richness of the weaker structure does suggest that a number of these levels do couple with the ZOB state, but *via* indirect couplings involving the two *e*′′ doorway states, *D*_11_*D*_18_*m* = 2 and 2*D*_14_*D*_18_*m* = 2, and the HT-induced *e*′ one, *D*_18_*D*_29_*m* = 2.

Under this scenario, the 2*D*_18_*m* = 1 bright state character would be expected to be “smeared” amongst the resulting vibrational eigenstates through this indirect coupling mechanism, and this is evinced in the ZEKE spectra in Fig. 2, and 2D-LIF spectra in Fig. 4 and 5. The activity of the doorway states, *D*_11_*D*_18_*m* = 2, 2*D*_14_*D*_18_*m* = 2 and *D*_18_*D*_29_*m* = 2 would also be affected in a similar manner, confirming their contributions to the coupled vibrational eigenstates. At first sight, the integrated traces of the 14_2_18_1_*m*_2_ and 18_1_29_1_*m*_2_ transitions appear to suggest that there is little to no coupling of these levels with the *D*_11_*D*_18_*m* = 2 level, owing to the absence of intensity in these traces at ∼850 cm^–1^; however, Fig. 6 shows horizontal slices of the 2D-LIF spectra which clearly shows that: (i) the appearance changes with the fluorescence wavenumber owing to accessing different rotational levels; and (ii) weak activity at ∼850 cm^–1^ is seen in some slices for both 18_1_29_1_*m*_2_ and 14_2_18_1_*m*_2_, indicating that all three doorway states have activity across this energy range. Together with the similarity in the peak positions in these slices across all three terminating states, 11_1_18_1_*m*_2_, 18_1_29_1_*m*_2_ and 14_2_18_1_*m*_2_, we conclude that coupling between the doorway states likely occurs. This will need to be confirmed through future higher-resolution 2D-LIF studies that can reveal the rotational structure in more detail.

We recall a similar doorway state scenario in toluene,^37^ where the ZEKE spectrum *via* one band (“Band U”) led to a completely unstructured ZEKE spectrum, while other spectra showed only the Δ*v* = 0 bands; we interpreted this as the upper level of the transition giving rise to “Band U” being strongly coupled to the bath states, and that its coupling to the other levels was the source of the broad underlying background for other bands.

We note that here, the 933 cm^–1^ ZEKE band assigned to 18^1^29^1^*m*^2^ is actually quite intense (see Fig. 2), while the corresponding band in the DF spectrum is relatively weak – this may be an effect of the different timescales for the two experiments, with ionization being very rapid compared to fluorescence. In addition, we note that the intensities are not consistent with the usual Δ(*v*,*m*) = 0 activity. For example the ZEKE band corresponding to 11^1^18^1^*m*^2^ is fairly weak throughout the spectra, but the 11^2^18^1^*m*^2^ band is as intense as the 11^1^18^2^, band, even though the 11^1^18^2^ band is significantly less intense than the 18^2^ band. Also of note is that the 18^1^*m*^2^ band at 509 cm^–1^ has significant intensity (note that this is in contrast to the more usual Δ(*v*,*m*) = 0 activity seen in the 2D-LIF spectrum – see above). Indeed, we note that part of the intensity of the 509 cm^–1^ band could arise from symmetry-allowed contributions from any eigenstate that arises from the mixing of the doorway states and the bath states, since each of the eigenstates will contain *D*_18_*m* = 2 character.

Our conclusion is thus that the involvement of the 2*D*_18_*m* = 0 level in energy dispersal is minimal, while that of the 2*D*_18_*m* = 1 level is significant.

Gascooke and Lawrance have discussed vibration-torsional coupling in detail in their work on toluene.^14^ The key points for the present work are:

(i) The efficiency of coupling is expected to decrease by about an order of magnitude for each vibrational quantum number change between the coupled states.^59^

(ii) Coupling between states with Δ*m* = 0 will be the strongest, with those between Δ*m* = 3 being significantly weaker, and those with Δ*m* = 6 weaker still^14^,^17^,^38^,^40^ (note that the coupling between states with Δ*m* = 0 may be viewed as simply anharmonic coupling, even though *m* ≠ 0 in some cases and hence represents coupling between vibtor levels).

The terms that cause vibration-torsion mixing between vibtor levels have one of the forms:^14^
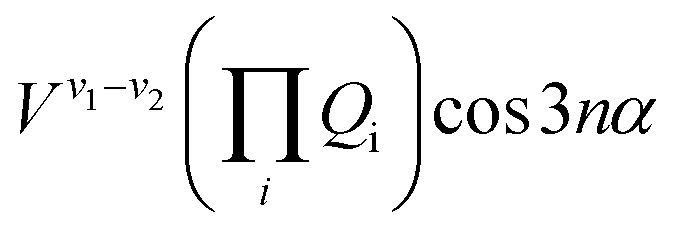
 
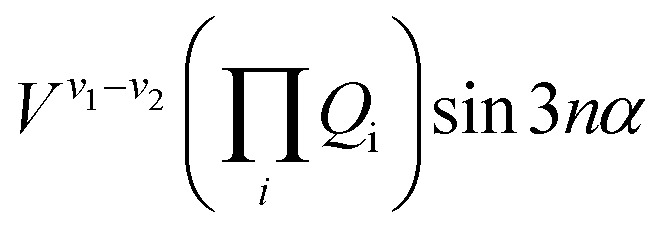
where the *V* terms are the coupling strengths, the *Q*_i_ are the normal mode coordinates, and *α* is the torsional angle; *n* takes integral values.

We first consider the contributing vibrations that give rise to the 400 cm^–1^ feature (*D*_29_, *D*_11_ and 2*D*_14_). We note that the interactions between each of *D*_11_ and 2*D*_14_ with *D*_29_ would be indirect *via* a HT interaction, as well as involving a *Q*_11_*Q*_29_ or *Q*_14_^2^*Q*_29_ coupling term, respectively. The *D*_11_ and 2*D*_14_ levels would be interacting *via* a *Q*_11_*Q*_14_^2^ term. The evidence from our ZEKE spectra is that there is at most weak coupling between these three levels.^38^ Further, we note that in *p*DFB^58^,^60^ the 14^2^ transition is fairly well separated in wavenumber from 11^1^ and so we do not expect the corresponding levels to be interacting strongly; from this we conclude that the 14^2^ and 11^1^ transitions can have their own optical brightness. Further, in the *p*-chlorofluorobenzene (*p*ClFB) molecule,^58^ the 11^1^ and 14^2^ transitions are almost coincident, suggesting strongly that the corresponding levels are not interacting to any significant extent, and supports the, at most, weak interaction between these levels.

We now focus on the ∼847 cm^–1^ feature, where there could be coupling involving the 2*D*_18_*m* = 1 level and the *D*_11_*D*_18_*m* = 2 and 2*D*_14_*D*_18_*m* = 2 levels (we have noted above that there cannot be a corresponding combination of the *D*_14_*m* = 6(–) level with a further vibtor level at 847 cm^–1^). The coupling between 2*D*_18_*m* = 1 and 2*D*_14_*D*_18_*m* = 2 will have the form *V*^18–2(14)^*Q*_14_^2^*Q*_18_ cos 3*α* and so be cubic in *Q*_i_ terms as well as involving a cos 3*α* term. Between *D*_11_*D*_18_*m* = 2 and 2*D*_14_*D*_18_*m* = 2, the interaction term will take the form *V*^11–2(14)^*Q*_11_*Q*_14_^2^ and so is cubic in *Q*_i_ terms, while between *D*_11_*D*_18_*m* = 2 and 2*D*_18_*m* = 1 it takes the form *V*^11–18^*Q*_11_*Q*_18_ cos 3*α* and so is quadratic in *Q*_i_ terms and also includes a cos 3*α* term, where the superscripts indicate the cardinal number of the interacting vibrational levels that are in combination with *D*_18_*m* = 2, with 2(14) representing 2*D*_14_. One might therefore expect the coupling between 2*D*_18_*m* = 1 and 2*D*_14_*D*_18_*m* = 2 to be the weakest as it is the highest order term (Δ*v* = 3, Δ*m* = 3). It is not immediately clear what the relative coupling strengths of *D*_11_*D*_18_*m* = 2 and 2*D*_18_*m* = 1 (Δ*v* = 2, Δ*m* = 3), and 2*D*_14_*D*_18_*m* = 2 and *D*_11_*D*_18_*m* = 2 (Δ*v* = 3) would be. A reasonable expectation is that since this is essentially an anharmonic coupling between the 2*D*_14_ and *D*_11_ levels, albeit each in combination with the *D*_18_*m* = 2 level, since that coupling is weak, then the interactions between the vibtor combinations might also expected to be weak, but caution is required as there may be effects caused by additional couplings (see below). Similarly, it is not immediately clear what the relative strength of coupling would be with the ZOS involving *D*_29_, whose activity is HT-induced.

The integrated intensity traces given in Fig. 5 give an insight to the degree of coupling between the ZOB state and the doorway states. Given the weakness of the integrated intensities of the traces shown in Fig. 5(d), (f) and (h), compared to those of the Δ(*v*,*m*) = 0 transitions in Fig. 5(e) and (g), the 2D-LIF spectra are dominated by the latter set of transitions. If each of the doorway states has no inherent oscillator strength, consistent with the non-observance of the 18^1^*m*^2^ transition in any of our excitation spectra, one may integrate across the whole feature in order to obtain an indication of the degree of coupling with the ZOB state; it is important to note that transitions terminating in the S_0_*D*_11_*D*_18_*m* = 2 and 2*D*_14_*D*_18_*m* = 2 levels are symmetry allowed from the S_1_ 2*D*_18_*m* = 1 level, whilst transitions to the *D*_18_*D*_29_*m* = 2 level may gain intensity through HT coupling, which we cannot disentangle from intensity resulting from coupling of the S_1_ levels. Overall, this results in relative intensities for the *D*_11_*D*_18_*m* = 2, *D*_18_*D*_29_*m* = 2 and 2*D*_14_*D*_18_*m* = 2 levels of 3.2, 1.4 and 1.0, respectively. The relative intensities of the *D*_11_*D*_18_*m* = 2 and 2*D*_14_*D*_18_*m* = 2 bands are consistent with expectations based on the changes in vibrational and torsional quanta discussed above, but also the intensity of the HT induced vibration-torsion coupling of the *D*_18_*D*_29_*m* = 2 band is significant. Notably, the larger value for the overall activity of *D*_11_*D*_18_*m* = 2 implies it is interacting the most strongly with 2*D*_18_*m* = 1, and this is consistent with the observed shift of the *m* = 1 component of the 18_2_ band to lower wavenumber.

The separation of the 14^2^ and 11^1^ features at 400 cm^–1^ is ∼10 cm^–1^, which is slightly greater than the spacing (7.5 cm^–1^) between the satellite bands in the ∼847 cm^–1^ feature. This is indicative of subtle changes in couplings between the ZOSs in the proximity ∼400 cm^–1^ and those at ∼847 cm^–1^.

We note that the *D*_14_*m* = 6(–) level was deduced in earlier work^38^ to be interacting with the 2*D*_14_ level, but it was incorrectly deduced that this led to two levels lying about 35 cm^–1^ apart, with one lying under the ∼400 cm^–1^ feature and one lying to lower wavenumber. This deduction arose from the appearance of a ZEKE band when exciting at 364 cm^–1^ that appeared to be consistent with its having 14^1^*m*^6(–)^ character, from the observation of a band which was at the same wavenumber as a feature seen when exciting close to 400 cm^–1^. We have now recorded 2D-LIF spectra over this region (not shown) that indicate that the lower wavenumber assignment is incorrect. However, those 2D-LIF spectra do confirm that that the *D*_14_*m* = 6(–) level does indeed lie underneath the band at ∼400 cm^–1^, consistent with our ZEKE study.^38^ Since this level is totally symmetric (*a*_2_′ × *a*_2_′ = *a*_1_′) it can potentially interact with the *D*_11_ and 2*D*_14_ levels; however, this effect appears to be extremely small. The slices of the 2D-LIF spectra in Fig. 6 also demonstrate that there is likely a rotational dependence of the coupling between each of these ZOSs; examining the ∼847 cm^–1^ region with higher resolution will likely aid interpretation of the complicated couplings in this energetic region.

In passing, we note that the *D*_11_*D*_18_*m* = 1 level should be at 839 cm^–1^ above the *m* = 0 state of the zero-point level in the S_1_ state. This would be of (*a*_1_′ × *a*_2_′′ × *e*′′=) *e*′ symmetry and so could interact with the *D*_18_*D*_29_*m* = 2 level (also *e*′ symmetry); however, no clear evidence for such interaction was seen.

### Comparison with time domain study

That only one of the lowest torsional levels is involved with close-to-dissipative energy dispersal is consistent with the observation made in ref. 33 that half of the 2*D*_18_ electron signal, covering both *m* = 0 and 1, was lost over time; however, we attribute this to the coupling of the 2*D*_18_*m* = 1 level with the bath states *via* the doorway states, rather than the corresponding *m* = 0 level, as concluded in that work. In passing, we note that one of the levels that was interacting with the ZOB was suggested^33^ as being the *D*_12_*m* = 6(–) vibtor level; however, we identified the 12^1^*m*^6(–)^ transition at different wavenumbers in both the S_1_ and D_0_^+^ states,^39^ and so we do not agree with the earlier assignment.^33^ Although we do concur with the conclusion from that work^33^ that a second doorway state level is interacting (a definitive assignment was not possible), here we have determined that three doorway states are involved in the coupling.

It is interesting to note that the energy differences between the interacting eigenstates were derived as 4.2 cm^–1^ and 6.7 cm^–1^ in ref. 33. In the present REMPI spectra (see Fig. 1), the separations between each satellite and the central band are measured as 2.8 cm^–1^ and 4.7 cm^–1^, with the satellites themselves separated by 7.5 cm^–1^ (see Fig. 1). Thus, the smaller separation determined in the time-resolved experiment appears to correspond to the separation of the eigenstates that arise from the pairwise interaction 2*D*_18_*m* = 1···*D*_11_*D*_18_*m* = 2, while the larger separation is consistent with those arising from 2*D*_14_*D*_18_*m* = 2···*D*_11_*D*_18_*m* = 2. The non-observation of the 2.8 cm^–1^ spacing seems to suggest that the interaction between 2*D*_14_*D*_18_*m* = 2 and 2*D*_18_*m* = 1 is weak, consistent with the higher-order dependence of the coupling noted above. Additionally, given the (at most) weak interaction between the *D*_11_ and 2*D*_14_ levels, noted above; this, and the results of the present work, suggests that the presence of torsional motion exacerbates the coupling between the 2*D*_14_*D*_18_*m* = 2 and *D*_11_*D*_18_*m* = 2 levels. This, and possible FC activity between the levels, confuses a clear interpretation of the details of coupling from the observed intensity profiles.

With regard to the assignment of the photoelectron bands employed for monitoring the time dependence of the wavepacket in ref. 33, the present deductions allow us to identify these as follows, noting that the resolution of the spectra (>30 cm^–1^) in ref. 33 means that a particular photoelectron band could contain more than one contribution (*cf.* the ZEKE spectra in Fig. 2). The 980 cm^–1^ feature (labelled band A in that work) was likely dominated by 18^2^ (both *m* = 0 and *m* = 1 components), but its lower wavenumber shoulder (visible in some traces) contains contributions from 11^1^18^1^*m*^2^ and 18^1^29^1^*m*^2^. The “in-phase” bands at 1415 cm^–1^ (band E) and 1805 cm^–1^ (band F) are likely to be 11^1^18^2^ and 9^1^18^2^ (and other unresolved bands). The out-of-phase 1200 cm^–1^ band B corresponds to 14^2^18^1^*m*^2^, confirming its identity as a doorway state, while the out-of-phase 1320 cm^–1^ band D seems to correspond to 9^1^18^1^*m*^2^ and so is in line with its activity arising from a dark state. The identity of the out-of-phase 890 cm^–1^ band C is unclear, but the ZEKE spectra in Fig. 2 suggest it has more than one component. One possibility is that this band is the convolution of the 9^1^*m*^3(+)^ and 29^2^*m*^3(+)^ bands, which were discussed above, and arise when exciting at position F in the present work (see Fig. 2). That the band is out-of-phase would be in line with HT coupling to the 2*D*_18_*m* = 0 level and so these states containing some dark state character, which would be consistent with the out-of-phase nature of this band.

## Summary

In the above, we have reassigned the main contributing levels that give rise to the REMPI feature at ∼847 cm^–1^. In so doing, we have uncovered direct evidence of the mechanism by which the methyl group, by virtue of excited torsional levels, can promote coupling between different vibrational energy levels of different symmetry and hence facilitate energy dispersal in a molecule. This is a more-general mechanism than that involving interactions between *a*_1_′ levels that has previously been reported.^14^,^17^,^35^,^38^–^41^ Although the role of the methyl group has been mooted for many years, the details of the mechanism have only become clear very recently, with Lawrance and Gascooke pioneering the investigation of vibration-torsion interactions. This work has unravelled couplings between torsions and vibtor levels, between torsional level-specific couplings within a Fermi resonance, and has hypothesised the importance of vibtor levels in vibrational energy dispersal. The difficulty in establishing the mechanism in the past has arisen owing to the complicated nature of the experiments, and so difficulty in controlling all parameters, and hence reliably interpreting the results; this can be further complicated by the unavailability of reliable wavenumbers and/or insufficient resolution to pick out the key bands and their positions. In the present work, we have benefited greatly from having previously identified reliable values for all of the out-of-plane vibrations of *p*FT in the S_1_ and D_0_^+^ states,^39^ with there being reasonably reliable values for all of the S_0_ ones,^49^ but some of which we have modified from our present and ongoing experiments – see Table 2. In addition, our resolution here is high enough to be able to identify individual vibrational bands clearly in both fluorescence and ZEKE experiments; extremely valuable is the correspondence of activity across the 2D-LIF and ZEKE spectra.

To the best of our knowledge, this is the first example of an unambiguous assignment of vibtor levels acting as doorway states, and thus enhancing vibrational energy dispersal. There is activity from 2*D*_18_ across the whole of the feature, supporting its being the ZOB state, with the evidence suggesting direct vibration-torsion coupling to two doorway states, and *via* HT coupling, to a third. These states also appear to be coupled to each other, as well as to the background bath of states. Future higher resolution studies of this coupling, resolving the rotational structure, are desirable. Of importance is that the corresponding 2*D*_18_*m* = 0 state has at most a very minor role in vibrational energy dispersal.

The methyl group is a very common substituent in biological molecules and so vibration-torsional coupling is expected to be of great importance in the facilitation of interactions between different vibrational motions and so the efficient, rapid dispersal of energy through such molecules.

## Conflicts of interest

There are no conflicts of interest.
